# Gliquidone

**DOI:** 10.1107/S1600536811016680

**Published:** 2011-05-07

**Authors:** Thomas Gelbrich, Mairi F. Haddow, Ulrich J. Griesser

**Affiliations:** a Institute of Pharmacy, University of Innsbruck, Innrain 52, 6020 Innsbruck, Austria

## Abstract

The title compound {systematic name: *N*-cyclo­hexyl­carba­moyl-4-[2-(7-meth­oxy-4,4-dimethyl-1,3-dioxo-1,2,3,4-tetra­hydro­isoquinolin-2-yl)eth­yl]benzene­sulfonamide}, C_27_H_33_N_3_O_6_S, displays an intra­molecular N—H⋯O=S inter­action, as well as inter­molecular N—H⋯O=C hydrogen bonds. The latter inter­actions lead to the formation of hydrogen-bonded chains parallel to the *c* axis. The conformation of the sulfonyl­urea fragment is in agreement with a recent theoretical study [Kasetti *et al.* (2010 ▶). *J. Phys. Chem. B*, **114**, 11603–11610].

## Related literature

For theoretical studies of the molecular structure, see Lins *et al.* (1996 ▶); Kasetti *et al.* (2010 ▶). For thermomicroscopy, see Kuhnert-Brandstätter *et al.* (1982 ▶). For related crystal structures, see: Kobelt & Paulus (1972 ▶); Iwata *et al.* (1997 ▶); Grell *et al.* (1998 ▶); Endo *et al.* (2003 ▶).
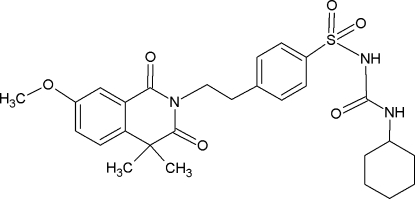


## Experimental

### 

#### Crystal data

C_27_H_33_N_3_O_6_S
*M* *_r_* = 527.62
Monoclinic, 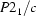

*a* = 19.1494 (4) Å
*b* = 10.7253 (3) Å
*c* = 13.8024 (2) Å
β = 106.691 (1)°
*V* = 2715.34 (10) Å^3^
*Z* = 4
Mo *K*α radiation
μ = 0.17 mm^−1^
*T* = 120 K
0.40 × 0.40 × 0.10 mm

#### Data collection

Bruker–Nonius Roper CCD camera on κ-goniostat diffractometer
Absorption correction: multi-scan (*SADABS*; Sheldrick, 2007 ▶) *T*_min_ = 0.937, *T*_max_ = 0.984
28737 measured reflections
5325 independent reflections
4361 reflections with *I* > 2σ(*I*)
*R*_int_ = 0.044

#### Refinement

*R*[*F* ^2^ > 2σ(*F* ^2^)] = 0.038
*wR*(*F* ^2^) = 0.101
*S* = 1.03
5325 reflections
376 parameters
2 restraints
H atoms treated by a mixture of independent and constrained refinement
Δρ_max_ = 0.28 e Å^−3^
Δρ_min_ = −0.43 e Å^−3^

### 

Data collection: *COLLECT* (Hooft, 1998 ▶); cell refinement: *DENZO* (Otwinowski & Minor, 1997 ▶) and *COLLECT*; data reduction: *DENZO* and *COLLECT*; program(s) used to solve structure: *SHELXS97* (Sheldrick, 2008 ▶); program(s) used to refine structure: *SHELXL97* (Sheldrick, 2008 ▶); molecular graphics: *XP* in *SHELXTL* (Sheldrick, 2008) ▶ and *Mercury* (Macrae *et al.*, 2006 ▶); software used to prepare material for publication: *publCIF* (Westrip, 2010 ▶).

## Supplementary Material

Crystal structure: contains datablocks I, global. DOI: 10.1107/S1600536811016680/jh2285sup1.cif

Structure factors: contains datablocks I. DOI: 10.1107/S1600536811016680/jh2285Isup2.hkl

Supplementary material file. DOI: 10.1107/S1600536811016680/jh2285Isup3.cml

Additional supplementary materials:  crystallographic information; 3D view; checkCIF report

## Figures and Tables

**Table 1:** Hydrogen-bond geometry (Å, °)

*D*—H⋯*A*	*D*—H	H⋯*A*	*D*⋯*A*	*D*—H⋯*A*
N1—H1*N*⋯O5^i^	0.86 (1)	2.03 (2)	2.8702 (17)	167 (2)
N2—H2*N*⋯O1	0.87 (1)	2.15 (2)	2.8404 (17)	136 (2)

Symmetry code: (i) 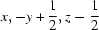.

**Fig. 1.**
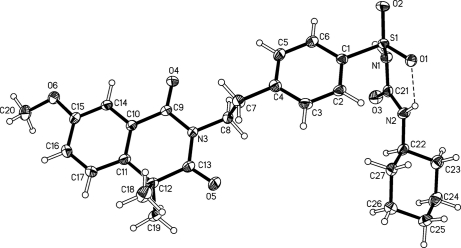
The molecular structure with displacement ellipsoids drawn at the 50% probability level. Hydrogen atoms are shown as spheres of arbitrary size.

**Fig. 2.**
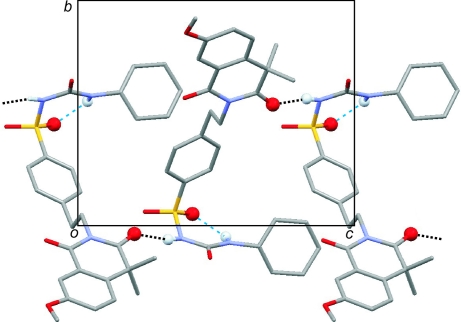
Fragment of an N–H···O=C-bonded chain. The crystal structure is viewed along [100]. H and O atoms directly engaged in H-bonds are drawn as balls.

## supplementary crystallographic information

### Comment

Gliquidone is an anti-diabetic drug in the sulfonylurea class. It is used in the treatment of diabetes mellitus type 2. The conformation of the sulfonyl urea fragment is in agreement with the SLU-1 geometry discussed in a recent theoretical study [Kasetti *et al.* (2010)]. The same conformation was previously found in polymorph I of glimepiride (Iwata *et al.*, 1997).

In the sulfonyl urea part, the molecule displays an intramolecular N–H···O=S bond. The second NH group is N–H···O=C bonded to the imide part of a neighbouring molecule. As a result of this interaction, H-bonded chains are formed, which possess glide symmetry and propagate parallel to the *c*-axis.

### Experimental

The investigated sample of gliquidone was obtained from Boehriger Ingelheim.

### Refinement

All H atoms were identified in a difference map. Methyl H atoms were idealized and included as rigid groups allowed to rotate but not tip (C—H = 0.98 Å). H atoms in CH_2_ (C—H = 0.99 Å) or CH (C—H = 1.00 Å) groups and H atoms bonded to aromatic carbon atoms (C—H = 0.95 Å) were positioned geometrically. Hydrogen atoms attached to N were refined with restrained distances [N—H = 0.88 (2) Å]. *U*_ĩso_ parameters for all H atoms were refined freely.

### Crystal data

**Table:**

C_27_H_33_N_3_O_6_S	*F*(000) = 1120
*M**_r_* = 527.62	*D*_x_ = 1.291 Mg m^−^^3^
Monoclinic, *P*2_1_/*c*	Mo *K*α radiation, λ = 0.71073 Å
Hall symbol: -P 2ybc	Cell parameters from 16476 reflections
*a* = 19.1494 (4) Å	θ = 2.9–27.5°
*b* = 10.7253 (3) Å	µ = 0.17 mm^−^^1^
*c* = 13.8024 (2) Å	*T* = 120 K
β = 106.691 (1)°	Plate, colourless
*V* = 2715.34 (10) Å^3^	0.40 × 0.40 × 0.10 mm
*Z* = 4	

### Data collection

**Table:**

Bruker–Nonius Roper CCD camera on κ-goniostat diffractometer	5325 independent reflections
Radiation source: Bruker-Nonius FR591 rotating anode	4361 reflections with *I* > 2σ(*I*)
graphite	*R*_int_ = 0.044
Detector resolution: 9.091 pixels mm^-1^	θ_max_ = 26.0°, θ_min_ = 2.9°
φ and ω scans	*h* = −23→23
Absorption correction: multi-scan (*SADABS*; Sheldrick, 2007)	*k* = −13→13
*T*_min_ = 0.937, *T*_max_ = 0.984	*l* = −15→17
28737 measured reflections	

### Refinement

**Table:**

Refinement on *F*^2^	Primary atom site location: structure-invariant direct methods
Least-squares matrix: full	Secondary atom site location: difference Fourier map
*R*[*F*^2^ > 2σ(*F*^2^)] = 0.038	Hydrogen site location: inferred from neighbouring sites
*wR*(*F*^2^) = 0.101	H atoms treated by a mixture of independent and constrained refinement
*S* = 1.03	*w* = 1/[σ^2^(*F*_o_^2^) + (0.054*P*)^2^ + 0.7288*P*] where *P* = (*F*_o_^2^ + 2*F*_c_^2^)/3
5325 reflections	(Δ/σ)_max_ = 0.001
376 parameters	Δρ_max_ = 0.28 e Å^−^^3^
2 restraints	Δρ_min_ = −0.43 e Å^−^^3^

### Special details

**Table:**

Geometry. All e.s.d.'s (except the e.s.d. in the dihedral angle between two l.s. planes) are estimated using the full covariance matrix. The cell e.s.d.'s are taken into account individually in the estimation of e.s.d.'s in distances, angles and torsion angles; correlations between e.s.d.'s in cell parameters are only used when they are defined by crystal symmetry. An approximate (isotropic) treatment of cell e.s.d.'s is used for estimating e.s.d.'s involving l.s. planes.
Refinement. Refinement of *F*^2^ against ALL reflections. The weighted *R*-factor *wR* and goodness of fit *S* are based on *F*^2^, conventional *R*-factors *R* are based on *F*, with *F* set to zero for negative *F*^2^. The threshold expression of *F*^2^ > σ(*F*^2^) is used only for calculating *R*-factors(gt) *etc*. and is not relevant to the choice of reflections for refinement. *R*-factors based on *F*^2^ are statistically about twice as large as those based on *F*, and *R*- factors based on ALL data will be even larger.

### Fractional atomic coordinates and isotropic or equivalent isotropic displacement parameters (Å^2^)

**Table:**

	*x*	*y*	*z*	*U*_iso_*/*U*_eq_	
S1	0.372978 (18)	0.06096 (4)	0.34758 (2)	0.02071 (11)	
O1	0.44637 (5)	0.05111 (11)	0.41217 (7)	0.0251 (2)	
O2	0.35950 (6)	0.06083 (11)	0.24022 (7)	0.0291 (3)	
O3	0.26601 (6)	−0.14284 (11)	0.47583 (8)	0.0301 (3)	
O4	0.04953 (6)	0.56657 (10)	0.38426 (7)	0.0264 (3)	
O5	0.19863 (6)	0.53754 (12)	0.70230 (8)	0.0384 (3)	
O6	−0.15069 (6)	0.85561 (11)	0.38996 (8)	0.0306 (3)	
N1	0.32644 (7)	−0.05733 (12)	0.37222 (9)	0.0229 (3)	
H1N	0.2868 (9)	−0.0629 (18)	0.3241 (13)	0.040 (5)*	
N2	0.38277 (7)	−0.07655 (13)	0.54610 (9)	0.0251 (3)	
H2N	0.4223 (8)	−0.0513 (17)	0.5327 (13)	0.031 (5)*	
N3	0.12334 (6)	0.55263 (11)	0.54472 (8)	0.0190 (3)	
C1	0.33329 (7)	0.19457 (14)	0.38350 (10)	0.0189 (3)	
C2	0.36549 (8)	0.24940 (15)	0.47723 (10)	0.0216 (3)	
H2	0.4099	0.2175	0.5203	0.027 (4)*	
C3	0.33191 (8)	0.35119 (15)	0.50681 (10)	0.0225 (3)	
H3	0.3534	0.3886	0.5709	0.034 (5)*	
C4	0.26712 (8)	0.39936 (14)	0.44388 (11)	0.0217 (3)	
C5	0.23624 (8)	0.34344 (15)	0.35000 (11)	0.0244 (3)	
H5	0.1922	0.3760	0.3065	0.031 (5)*	
C6	0.26863 (8)	0.24152 (15)	0.31929 (11)	0.0228 (3)	
H6	0.2471	0.2040	0.2553	0.031 (4)*	
C7	0.22896 (8)	0.50595 (15)	0.47875 (11)	0.0248 (3)	
H7A	0.2106	0.5662	0.4228	0.040 (5)*	
H7B	0.2635	0.5500	0.5358	0.026 (4)*	
C8	0.16539 (8)	0.45372 (14)	0.51243 (11)	0.0215 (3)	
H8A	0.1325	0.4065	0.4558	0.026 (4)*	
H8B	0.1845	0.3950	0.5692	0.031 (5)*	
C9	0.06399 (7)	0.60349 (14)	0.47150 (10)	0.0184 (3)	
C10	0.02037 (7)	0.69887 (13)	0.50490 (10)	0.0184 (3)	
C11	0.04048 (8)	0.74227 (14)	0.60447 (10)	0.0214 (3)	
C12	0.10805 (8)	0.69515 (14)	0.68274 (10)	0.0216 (3)	
C13	0.14635 (8)	0.58977 (15)	0.64517 (11)	0.0232 (3)	
C14	−0.04261 (7)	0.74093 (14)	0.43406 (10)	0.0204 (3)	
H14	−0.0546	0.7117	0.3663	0.026 (4)*	
C15	−0.08755 (8)	0.82517 (15)	0.46246 (11)	0.0235 (3)	
C16	−0.06813 (9)	0.87156 (16)	0.56085 (11)	0.0301 (4)	
H16	−0.0983	0.9310	0.5803	0.038 (5)*	
C17	−0.00459 (9)	0.83075 (16)	0.63026 (11)	0.0290 (4)	
H17	0.0086	0.8638	0.6969	0.035 (5)*	
C18	0.16458 (9)	0.80078 (17)	0.71456 (13)	0.0368 (4)	
H18A	0.1814	0.8253	0.6566	0.054 (6)*	
H18B	0.2061	0.7716	0.7695	0.044 (5)*	
H18C	0.1421	0.8727	0.7378	0.042 (5)*	
C19	0.08837 (9)	0.64511 (17)	0.77617 (11)	0.0331 (4)	
H19A	0.0675	0.7126	0.8069	0.047 (6)*	
H19B	0.1324	0.6133	0.8254	0.039 (5)*	
H19C	0.0526	0.5776	0.7556	0.068 (7)*	
C20	−0.20530 (9)	0.91933 (18)	0.42316 (13)	0.0369 (4)	
H20A	−0.2180	0.8696	0.4752	0.042 (5)*	
H20B	−0.2488	0.9314	0.3657	0.047 (6)*	
H20C	−0.1865	1.0006	0.4513	0.039 (5)*	
C21	0.32254 (8)	−0.09519 (14)	0.46937 (11)	0.0217 (3)	
C22	0.38491 (8)	−0.09235 (15)	0.65249 (11)	0.0228 (3)	
H22	0.3337	−0.1030	0.6557	0.031 (4)*	
C23	0.42853 (9)	−0.20722 (15)	0.69978 (12)	0.0292 (4)	
H23A	0.4786	−0.2015	0.6932	0.029 (4)*	
H23B	0.4053	−0.2830	0.6636	0.049 (6)*	
C24	0.43184 (9)	−0.21684 (16)	0.81155 (12)	0.0333 (4)	
H24A	0.3821	−0.2302	0.8174	0.041 (5)*	
H24B	0.4619	−0.2897	0.8419	0.051 (6)*	
C25	0.46396 (9)	−0.10010 (17)	0.86938 (12)	0.0328 (4)	
H25A	0.5152	−0.0906	0.8687	0.032 (5)*	
H25B	0.4636	−0.1082	0.9407	0.039 (5)*	
C26	0.42077 (9)	0.01468 (17)	0.82278 (12)	0.0314 (4)	
H26A	0.4446	0.0901	0.8588	0.035 (5)*	
H26B	0.3710	0.0096	0.8304	0.039 (5)*	
C27	0.41609 (8)	0.02515 (15)	0.71081 (11)	0.0251 (3)	
H27A	0.3849	0.0972	0.6812	0.031 (5)*	
H27B	0.4654	0.0407	0.7039	0.027 (4)*	

### Atomic displacement parameters (Å^2^)

**Table:**

	*U*^11^	*U*^22^	*U*^33^	*U*^12^	*U*^13^	*U*^23^
S1	0.01746 (19)	0.0296 (2)	0.01411 (19)	0.00384 (15)	0.00301 (14)	−0.00118 (14)
O1	0.0158 (5)	0.0382 (7)	0.0206 (5)	0.0056 (4)	0.0038 (4)	0.0007 (4)
O2	0.0295 (6)	0.0427 (7)	0.0148 (5)	0.0065 (5)	0.0059 (4)	−0.0014 (5)
O3	0.0242 (6)	0.0330 (7)	0.0310 (6)	−0.0057 (5)	0.0048 (4)	−0.0019 (5)
O4	0.0293 (6)	0.0314 (6)	0.0161 (5)	0.0073 (5)	0.0026 (4)	−0.0029 (4)
O5	0.0346 (7)	0.0451 (8)	0.0252 (6)	0.0187 (6)	−0.0080 (5)	−0.0040 (5)
O6	0.0235 (6)	0.0404 (7)	0.0238 (5)	0.0148 (5)	0.0003 (4)	0.0004 (5)
N1	0.0215 (7)	0.0252 (7)	0.0183 (6)	0.0010 (5)	0.0000 (5)	−0.0041 (5)
N2	0.0173 (6)	0.0384 (8)	0.0185 (6)	0.0005 (6)	0.0033 (5)	0.0028 (5)
N3	0.0191 (6)	0.0196 (6)	0.0169 (6)	0.0035 (5)	0.0029 (5)	0.0004 (5)
C1	0.0166 (7)	0.0237 (8)	0.0166 (7)	−0.0004 (6)	0.0054 (5)	0.0004 (6)
C2	0.0157 (7)	0.0307 (8)	0.0164 (7)	−0.0004 (6)	0.0014 (5)	0.0014 (6)
C3	0.0219 (7)	0.0284 (8)	0.0166 (7)	−0.0022 (6)	0.0045 (6)	−0.0036 (6)
C4	0.0207 (7)	0.0228 (8)	0.0229 (7)	−0.0010 (6)	0.0085 (6)	0.0005 (6)
C5	0.0190 (7)	0.0285 (9)	0.0219 (7)	0.0036 (6)	−0.0002 (6)	0.0010 (6)
C6	0.0201 (7)	0.0268 (8)	0.0182 (7)	0.0006 (6)	0.0002 (6)	−0.0019 (6)
C7	0.0242 (8)	0.0250 (8)	0.0257 (8)	0.0009 (6)	0.0078 (6)	−0.0015 (6)
C8	0.0211 (7)	0.0204 (8)	0.0216 (7)	0.0043 (6)	0.0042 (6)	0.0003 (6)
C9	0.0183 (7)	0.0198 (7)	0.0161 (7)	−0.0006 (6)	0.0032 (5)	0.0016 (6)
C10	0.0193 (7)	0.0182 (7)	0.0166 (7)	−0.0001 (6)	0.0034 (5)	0.0012 (5)
C11	0.0238 (8)	0.0219 (8)	0.0166 (7)	0.0007 (6)	0.0024 (6)	0.0002 (6)
C12	0.0242 (7)	0.0216 (8)	0.0161 (7)	0.0021 (6)	0.0009 (6)	−0.0008 (6)
C13	0.0206 (7)	0.0270 (8)	0.0185 (7)	0.0018 (6)	−0.0001 (6)	0.0003 (6)
C14	0.0208 (7)	0.0237 (8)	0.0153 (7)	0.0013 (6)	0.0031 (5)	0.0008 (6)
C15	0.0211 (7)	0.0254 (8)	0.0212 (7)	0.0060 (6)	0.0015 (6)	0.0032 (6)
C16	0.0332 (9)	0.0292 (9)	0.0261 (8)	0.0125 (7)	0.0056 (7)	−0.0037 (7)
C17	0.0347 (9)	0.0293 (9)	0.0193 (7)	0.0091 (7)	0.0018 (6)	−0.0046 (6)
C18	0.0340 (9)	0.0289 (9)	0.0375 (9)	−0.0039 (8)	−0.0057 (7)	−0.0019 (7)
C19	0.0365 (9)	0.0412 (10)	0.0205 (8)	0.0113 (8)	0.0064 (7)	0.0078 (7)
C20	0.0274 (9)	0.0439 (11)	0.0387 (10)	0.0177 (8)	0.0084 (7)	0.0050 (8)
C21	0.0221 (8)	0.0189 (7)	0.0229 (7)	0.0044 (6)	0.0045 (6)	−0.0022 (6)
C22	0.0169 (7)	0.0304 (9)	0.0207 (7)	0.0018 (6)	0.0048 (6)	0.0058 (6)
C23	0.0310 (9)	0.0233 (8)	0.0313 (8)	0.0007 (7)	0.0059 (7)	0.0023 (7)
C24	0.0335 (9)	0.0319 (9)	0.0333 (9)	0.0044 (7)	0.0074 (7)	0.0139 (7)
C25	0.0330 (9)	0.0425 (10)	0.0210 (8)	0.0055 (8)	0.0044 (6)	0.0052 (7)
C26	0.0348 (9)	0.0357 (10)	0.0246 (8)	0.0082 (8)	0.0101 (7)	0.0001 (7)
C27	0.0264 (8)	0.0242 (8)	0.0251 (8)	0.0061 (6)	0.0078 (6)	0.0040 (6)

### Geometric parameters (Å, °)

**Table:**

S1—O2	1.4293 (10)	C11—C12	1.5146 (19)
S1—O1	1.4357 (10)	C12—C13	1.517 (2)
S1—N1	1.6413 (14)	C12—C19	1.540 (2)
S1—C1	1.7588 (15)	C12—C18	1.541 (2)
O3—C21	1.2234 (18)	C14—C15	1.380 (2)
O4—C9	1.2213 (17)	C14—H14	0.9500
O5—C13	1.2184 (18)	C15—C16	1.393 (2)
O6—C15	1.3692 (17)	C16—C17	1.386 (2)
O6—C20	1.4305 (19)	C16—H16	0.9500
N1—C21	1.4233 (19)	C17—H17	0.9500
N1—H1N	0.856 (14)	C18—H18A	0.9800
N2—C21	1.3380 (19)	C18—H18B	0.9800
N2—C22	1.4670 (18)	C18—H18C	0.9800
N2—H2N	0.872 (14)	C19—H19A	0.9800
N3—C13	1.3871 (18)	C19—H19B	0.9800
N3—C9	1.3968 (18)	C19—H19C	0.9800
N3—C8	1.4759 (18)	C20—H20A	0.9800
C1—C2	1.393 (2)	C20—H20B	0.9800
C1—C6	1.3935 (19)	C20—H20C	0.9800
C2—C3	1.387 (2)	C22—C27	1.522 (2)
C2—H2	0.9500	C22—C23	1.526 (2)
C3—C4	1.393 (2)	C22—H22	1.0000
C3—H3	0.9500	C23—C24	1.529 (2)
C4—C5	1.396 (2)	C23—H23A	0.9900
C4—C7	1.508 (2)	C23—H23B	0.9900
C5—C6	1.382 (2)	C24—C25	1.517 (2)
C5—H5	0.9500	C24—H24A	0.9900
C6—H6	0.9500	C24—H24B	0.9900
C7—C8	1.529 (2)	C25—C26	1.519 (2)
C7—H7A	0.9900	C25—H25A	0.9900
C7—H7B	0.9900	C25—H25B	0.9900
C8—H8A	0.9900	C26—C27	1.526 (2)
C8—H8B	0.9900	C26—H26A	0.9900
C9—C10	1.476 (2)	C26—H26B	0.9900
C10—C14	1.3916 (19)	C27—H27A	0.9900
C10—C11	1.3963 (19)	C27—H27B	0.9900
C11—C17	1.396 (2)		
			
O2—S1—O1	119.79 (6)	O6—C15—C14	116.12 (13)
O2—S1—N1	105.48 (7)	O6—C15—C16	124.05 (14)
O1—S1—N1	107.90 (7)	C14—C15—C16	119.82 (13)
O2—S1—C1	109.21 (7)	C17—C16—C15	119.79 (14)
O1—S1—C1	108.07 (7)	C17—C16—H16	120.1
N1—S1—C1	105.49 (7)	C15—C16—H16	120.1
C15—O6—C20	116.95 (12)	C16—C17—C11	121.52 (14)
C21—N1—S1	126.46 (10)	C16—C17—H17	119.2
C21—N1—H1N	115.9 (13)	C11—C17—H17	119.2
S1—N1—H1N	107.8 (13)	C12—C18—H18A	109.5
C21—N2—C22	123.02 (13)	C12—C18—H18B	109.5
C21—N2—H2N	118.8 (11)	H18A—C18—H18B	109.5
C22—N2—H2N	118.1 (11)	C12—C18—H18C	109.5
C13—N3—C9	124.75 (12)	H18A—C18—H18C	109.5
C13—N3—C8	117.64 (11)	H18B—C18—H18C	109.5
C9—N3—C8	117.60 (11)	C12—C19—H19A	109.5
C2—C1—C6	120.89 (14)	C12—C19—H19B	109.5
C2—C1—S1	119.59 (11)	H19A—C19—H19B	109.5
C6—C1—S1	119.48 (11)	C12—C19—H19C	109.5
C3—C2—C1	119.12 (13)	H19A—C19—H19C	109.5
C3—C2—H2	120.4	H19B—C19—H19C	109.5
C1—C2—H2	120.4	O6—C20—H20A	109.5
C2—C3—C4	120.90 (13)	O6—C20—H20B	109.5
C2—C3—H3	119.6	H20A—C20—H20B	109.5
C4—C3—H3	119.6	O6—C20—H20C	109.5
C3—C4—C5	118.90 (14)	H20A—C20—H20C	109.5
C3—C4—C7	120.55 (13)	H20B—C20—H20C	109.5
C5—C4—C7	120.47 (13)	O3—C21—N2	125.76 (14)
C6—C5—C4	121.11 (13)	O3—C21—N1	118.35 (13)
C6—C5—H5	119.4	N2—C21—N1	115.88 (13)
C4—C5—H5	119.4	N2—C22—C27	109.13 (12)
C5—C6—C1	119.07 (13)	N2—C22—C23	112.08 (12)
C5—C6—H6	120.5	C27—C22—C23	111.03 (12)
C1—C6—H6	120.5	N2—C22—H22	108.2
C4—C7—C8	108.68 (12)	C27—C22—H22	108.2
C4—C7—H7A	110.0	C23—C22—H22	108.2
C8—C7—H7A	110.0	C22—C23—C24	110.18 (13)
C4—C7—H7B	110.0	C22—C23—H23A	109.6
C8—C7—H7B	110.0	C24—C23—H23A	109.6
H7A—C7—H7B	108.3	C22—C23—H23B	109.6
N3—C8—C7	112.30 (12)	C24—C23—H23B	109.6
N3—C8—H8A	109.1	H23A—C23—H23B	108.1
C7—C8—H8A	109.1	C25—C24—C23	111.60 (13)
N3—C8—H8B	109.1	C25—C24—H24A	109.3
C7—C8—H8B	109.1	C23—C24—H24A	109.3
H8A—C8—H8B	107.9	C25—C24—H24B	109.3
O4—C9—N3	119.59 (13)	C23—C24—H24B	109.3
O4—C9—C10	122.97 (12)	H24A—C24—H24B	108.0
N3—C9—C10	117.42 (12)	C24—C25—C26	110.91 (13)
C14—C10—C11	121.46 (13)	C24—C25—H25A	109.5
C14—C10—C9	117.34 (12)	C26—C25—H25A	109.5
C11—C10—C9	121.18 (12)	C24—C25—H25B	109.5
C17—C11—C10	117.50 (13)	C26—C25—H25B	109.5
C17—C11—C12	120.60 (12)	H25A—C25—H25B	108.0
C10—C11—C12	121.90 (13)	C25—C26—C27	110.78 (13)
C11—C12—C13	113.74 (11)	C25—C26—H26A	109.5
C11—C12—C19	110.43 (12)	C27—C26—H26A	109.5
C13—C12—C19	106.57 (12)	C25—C26—H26B	109.5
C11—C12—C18	110.13 (13)	C27—C26—H26B	109.5
C13—C12—C18	106.03 (13)	H26A—C26—H26B	108.1
C19—C12—C18	109.78 (13)	C22—C27—C26	112.00 (13)
O5—C13—N3	118.75 (14)	C22—C27—H27A	109.2
O5—C13—C12	120.60 (13)	C26—C27—H27A	109.2
N3—C13—C12	120.63 (12)	C22—C27—H27B	109.2
C15—C14—C10	119.86 (13)	C26—C27—H27B	109.2
C15—C14—H14	120.1	H27A—C27—H27B	107.9
C10—C14—H14	120.1		

### Hydrogen-bond geometry (Å, °)

**Table:**

*D*—H···*A*	*D*—H	H···*A*	*D*···*A*	*D*—H···*A*
N1—H1N···O5^i^	0.86 (1)	2.03 (2)	2.8702 (17)	167 (2)
N2—H2N···O1	0.87 (1)	2.15 (2)	2.8404 (17)	136 (2)

Symmetry codes: (i) *x*, −*y*+1/2, *z*−1/2.
